# Effect of PbSO_4_-Oleate Coverage on Cesium Lead Halide Perovskite Quantum Dots to Control Halide Exchange Kinetics

**DOI:** 10.3390/nano11102515

**Published:** 2021-09-27

**Authors:** Yeonsu Woo, Seeun Park, Seog Joon Yoon

**Affiliations:** Department of Chemistry, College of Natural Science, Yeungnam University, 280 Daehak-Ro, Gyeongbuk, Gyeongsan 38541, Korea; dndustn19@naver.com (Y.W.); qqkrtp1220@naver.com (S.P.)

**Keywords:** perovskite, perovskite PQDs, in situ spectroscopy, halide exchange

## Abstract

The selective control of halide ion exchange in metal halide perovskite quantum dots (PQDs) plays an important role in determining their band gap and composition. In this study, CsPbX_3_ (X = Cl^−^, Br^−^, and I^−^) PQDs were self-assembled with PbSO_4_-oleate to form a peapod-like morphology to selectively control halide ion exchange. Considering the distinct absorption and bright luminescence characteristics of these PQDs, in situ UV-Vis. absorption and fluorescence spectroscopies were employed to monitor the time-dependent band gap and compositional changes of the PQDs. We determined that the halide exchange in the capped PQDs is hindered—unlike the rapid anion exchange in noncapped PQDs—by a reduction in the halide exchange kinetic rate depending on the extent of coverage of the PQDs. Thus, we tracked the halide ion exchange kinetics between CsPbBr_3_ and CsPbI_3_ PQDs, depending on the coverage, using in situ UV-Vis. absorption/photoluminescence spectroscopy. We regulated the halide exchange reaction rate by varying the capping reaction temperature of the PQDs. The capping hindered the halide exchange kinetics and increased the activation energy. These results will enable the development of white LEDs, photovoltaic cells, and photocatalysts with alternative structural designs based on the divalent composition of CsPbX_3_ PQDs.

## 1. Introduction

All-inorganic cesium lead halide (CsPbX_3_, X: Cl, Br, or I) perovskite quantum dots (PQDs) have emerged as actively studied materials owing to their distinct photophysical properties such as high photoluminescence quantum yields (PLQYs, >90%) [1], dominant radiative recombination processes [1,2,3] and band gap tunability [4,5]. These outstanding properties have promoted the application of PQDs in various optoelectronic devices and applications such as lasing [6], nonlinear optics [7], light-emitting diodes [4], solar cells [8], and solar-driven chemistry-based devices [1]. PQD size can be controlled by varying the band gap; however, the halide composition plays a more important role in achieving extensive band gap tunability in the range of 410–700 nm [5]. Notably, valence band maximum (VBM) energy level formation predominantly entails halide p-orbitals. By contrast, Pb p-orbitals mainly contribute to determining the energy level of the conduction band minimum (CBM). Other A-site or B-site compositions could be employed to vary the energy levels of the CBM/VBM; nevertheless, X-site halides are mainly used to control the energy levels and band gap [9]. Based on the synthetic procedure for PQDs, the halide composition can be controlled by varying divalent halide precursor ratios [1,4]. Post-synthetic halide exchange can also be applied to control the band gap of PQDs [4]. Of these two synthetic and postsynthetic processes, the halide exchange process can be employed more easily and provides greater band gap tunability based on a fine mixture of two different CsPbBr_3_/CsPbI_3_ (or CsPbCl_3_/CsPbBr_3_) PQDs. This is because halide migration in the perovskite crystalline domain and thermodynamically favorable mixing processes occur between the two different PQDs [10]. Numerous halide migration pathways have been proposed, such as migration through Schottky defects, Frenkel defects, lattice distortion by accumulated charges/impurities, grain boundary channels, lattice softening by lamination, or strain-induced extended defects arising from the piezoelectric effect [11]. Among the aforementioned possible pathways, vacancy-mediated halide migration could provide a favorable migration process because of the lower activation energy required to initiate halide migration [12,13]. The halide vacancies are also an important factor in determining the PLQYs and photocatalytic activities of the PQDs [1]. If a halide-deficient perovskite can absorb photons and generate photoinduced exciton/free carriers, such carriers in the PQDs can be trapped at the halide vacancies and the trapped electrons can undergo a non-radiative recombination process [1,2,3]. If the nonradiative recombination process is more dominant than the radiative recombination process, a low PLQY would be obtained, and the performance of the optoelectronic devices based on such PQDs would also be limited [14].

Halide migration in perovskites can be useful for tuning the band gap. However, such migration is sometimes detrimental to the performance and stability of perovskite-based devices owing to hysteresis [15] and phase segregation [4,13]. Furthermore, to obtain white light emission by mixing different halide PQDs, halide migration between the different PQDs must be prohibited. If the PQDs are mixed without effective surface passivation [1] or an insulating capping layer/shell [16], the white emission would be lost, and single-wavelength emission would be attained as a result of halide exchange among the PQDs. Efficient ligand-based surface passivation using various ligands can be adopted to hinder direct contact between the perovskites based on electrostatic stabilization and steric stabilization. In addition, an insulated SiO_2_ shell can be introduced on the perovskite core to block halide migration [17]. Recently, Kamat et al. reported that PbSO_4_-oleate coverage on top of PQD surfaces significantly impedes the halide exchange process for up to 3 h [18]. By forming a peapod-like morphology, the PbSO_4_-oleate capping on the perovskites enabled white emission from a mixed-PQD solution [19]. This achievement demonstrates the potential for applying PQDs in light-emitting devices as well as in tandem layers for solar cell applications. To study the retardation of the halide exchange kinetics, it is necessary to understand the changes in the kinetics with respect to the surrounding temperature to obtain the activation energy, as described by the Arrhenius equation:

(1) $$k=Ae^{\frac{-E_{a}}{RT}}$$

where *k*, *A*, *E_a_*, *R*, and *T* represent the kinetic rate constant, Arrhenius constant, activation energy, gas constant, and temperature, respectively. By varying the temperature of two different PbSO_4_-oleate-capped CsPbBr_3_ and CsPbI_3_ PQD solutions, we monitored the halide exchange kinetics through in situ UV-Vis. absorption and photoluminescence (PL) spectroscopy. Through the two techniques, we confirmed that capping significantly increases the activation energy of the halide exchange kinetics. This finding could provide deeper insights into the precise control of the halide exchange process.

## 2. Materials and Methods

### 2.1. Materials

Cesium carbonate (Cs_2_CO_3_, Samchun Chemicals, Seoul, Korea, 534-17-8, 99.5%), oleic acid (OA, Alfa aesar, Haverhill, MA, USA, 112-80-1, 90%), 1-octadecene (1-ODE, Sigma-Aldrich, Saint Louis, MI, USA, 112-88-9, 90%), PbI_2_ (Aldrich, 10101-63-0, 99%), PbBr_2_ (Alfa aesar, Haverhill, MA, USA, 10031-22-8, 98%), oleylamine (OAm, TCI, Paris, France, 112-90-3, 50%), n-hexane (Daejung, Siheung-si, Korea, 4081-2304, 95%), methyl acetate (MeOAc, Daejung, Siheung-si, Korea, 5555-4105, 99.5%), tetrabutylammonium hydrogen sulfate (TBAHS, Daejung, Siheung-si, Korea, 207-09732, 98%), acetone (Daejung, Siheung-si, Korea, 1009-4410, 99.5%), chloroform (Samchun Chemicals, Seoul, Korea, 000C0583, 99.5%), ethanol (Samchun Chemicals, Seoul, Korea, 000E0219, 95%), PbCl_2_ (Daejung, Siheung-si, Korea, 5076-4405, 98%), and riboflavin (Daejung, Siheung-si, Korea, 83-88-5, 98%) were used to synthesize the CsPbX_3_ (X = Br or I) PQDs and PbSO_4_-oleate clusters and for the subsequent analyses. Further, 1-octadecene was heated at 120 °C for 2 h to remove dissolved oxygen. In the case of hexane, acetone, and ethanol, 4 Å, 4–8 mesh molecular sieves were used to eliminate water in the solvent, and N_2_ purging was performed to stabilize the synthesis of the PQDs and PbSO_4_-oleate clusters.

### 2.2. Synthesis and Purification of CsPbX_3_ (X = Br or I) PQDs

The CsPbX_3_ PQDs were synthesized according to previously reported procedures, with modifications [2,4,5,20]. In brief, Cs-oleate was mixed with 0.4 g of Cs_2_CO_3_, 20 mL of 1-ODE, and 1.25 mL of OA in a 50 mL three-neck flask under vacuum at 120 °C for 30 min with stirring. Next, the flask was subjected to N_2_ purging and heated to 140 °C until a clear solution was obtained. Before performing the hot-injection process, the interior temperature of the Cs-oleate-containing flask was maintained at 115–120 °C under an N_2_ atmosphere. To synthesize CsPbX_3_ PQDs, 0.5 g of PbI_2_ or 0.4 g of PbBr_2_ and 25 mL of 1-ODE were mixed in a 100 mL three-neck flask under vacuum at 120 °C for 30 min with stirring. Concurrently, OA and OAm (2.5 mL) were mixed and placed into a 50 mL beaker and heated at 130 °C until the reaction was complete (i.e., until a yellow color was observed). The vacuum applied to the aforenoted three-neck flask was gently switched to an N_2_ atmosphere; subsequently, the heated OA-OAm solution was added to another flask. The N_2_ atmosphere was then switched to a vacuum to remove bubbles inside the aforenoted three-neck flask for ~5 min until a clear solution with dissolved PbI_2_ or PbBr_2_ was observed. This reaction flask was then purged with N_2_ and heated at 170 °C. When the reaction temperature was reached, 2 mL of the Cs-oleate solution was swiftly injected into the reaction flask. After 5 s, the reaction flask was dipped into an ice bath to quench the NC growth. For purification, 5 mL of 1-ODE was added to the reaction flask and centrifuged at 7000 rpm for 10 min. The supernatant was discarded, and the pellets were washed with 2 mL hexane and 2 mL MeOAc, and then centrifuged at 7000 rpm for 10 min. After the pellets were completely resuspended in 6 mL hexane, the solution was centrifuged again at 4000 rpm for 5 min to remove impurities. The pellets were discarded, and the stable supernatant was retained.

### 2.3. Synthesis of PbSO_4_-Oleate-Capped CsPbX_3_ PQDs

The PbSO_4_-oleate solution was synthesized as described elsewhere [18,19]. In short, 0.139 g of PbCl_2_ and 8 mL of OAm were added to a 20 mL vial and heated at 165 °C for 10 min with stirring on a hot plate. After 10 min, OA (0.5 mL) was added to the vial at 165 °C for 10 min with stirring and cooled to room temperature. A stock solution was prepared by adding 0.424 g of TBAHS to 10 mL of acetone. PbSO_4_-oleate clusters were obtained by mixing the following reactants into a 50 mL centrifuge tube: a 3.2 mL Pb-oleate solution, a 1.6 mL stock solution, 3.2 mL of OA, and 10 mL of chloroform. The mixture was shaken to dissolve the reactants. Then, 30 mL of anhydrous ethanol (as the antisolvent) was added to obtain the precipitate. The solution was centrifuged at 7000 rpm for 5 min, and white pellets were obtained. The white pellets were dried in a desiccator overnight and then suspended in 2 mL chloroform. To obtain PbSO_4_-oleate-capped CsPbX_3_, 6 mL of the purified CsPbX_3_ PQD dispersed solution in hexane was added to 2 mL PbSO_4_-oleate clusters in chloroform under dark conditions at 4 °C. To purify the capped CsPbX_3_ PQDs, the solution was centrifuged at 1500 rpm for 15 min. The supernatant substantially uncapped CsPbX_3_; thus, the supernatant was discarded. The remaining pellets (capped PQDs) were redispersed in 6 mL hexane.

### 2.4. Measurement of Photoluminescence Quantum Yield of PQDs

The PLQY of the PQDs was calculated by employing an ethanol solution with dissolved riboflavin dye as a reference. The PQDs were dispersed in hexane. The absorbance of riboflavin and the PQDs was maintained at 0.1 for a 430 nm wavelength. The PLQY calculation was performed according to the literature [2,21] as follows:

$$\phi_{sample}=\phi_{standard}\times \frac{Area_{sample}}{Area_{standard}}\times \frac{\eta_{sample}^{2}}{\eta_{standard}^{2}}$$

where $\phi_{sample}$ = unknown PLQY of sample,

$\phi_{standard}$ = known PLQY of reference (in this case, for riboflavin absorbance of 0.1 at 430 nm),
$Area_{sample}$ = integrated PL intensity of sample,
$Area_{standard}$ = integrated PL intensity of standard,
$\eta_{sample}$ = refractive index of the solvent in which the sample was dispersed (in this case, 1.375 for hexane), and
$\eta_{standard}$ = refractive index of the solvent in which the reference was dispersed (in this case, 1.361 for ethanol).

### 2.5. Characterizations

TEM images were acquired using a Tecnai G2 F20 X-Twin microscope (FEI Korea, Hillsboro, OR, USA). XRD was performed using a D8 Advance diffractometer (Bruker, Billerica, MA, USA). An X-ray diffractometer equipped with a Cu Kα radiation source was employed in the 2θ range of 10–80° at 0.05 deg/step and 0.5 sec/step. In situ UV-Vis. absorption and photoluminescence spectroscopies were performed using an IPCE-based homemade optical setup (IPCE-CCD) with Duetta (HORIBA Scientific, Kyoto, Japan) as a charge-coupled device (CCD) detector. We used a 380 nm excitation light to excite the PQDs to prevent the occurrence of overtones up to 800 nm and to obtain pristine PL spectra of the PQDs.

## 3. Results and Discussions

### 3.1. Effect of PbSO_4_-Oleate Capping on Photophysical and Material Properties of CsPbX_3_ PQDs

**Figure 1:**
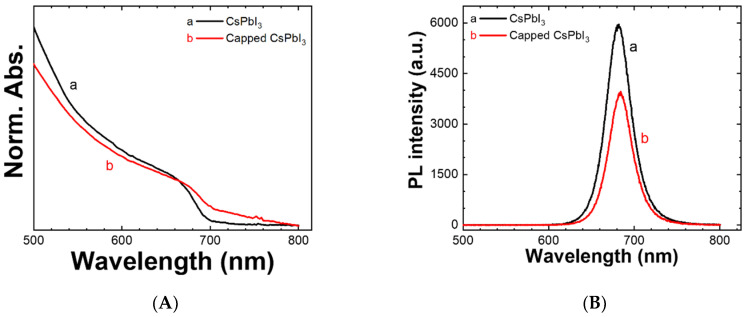
(**A**) Normalized UV-Vis. absorption spectra (normalized absorbance at band edge) of (a) pristine CsPbI_3_ PQDs without the peapod capping and (b) PbSO_4_-oleate-capped CsPbI_3_ PQDs. (**B**) Photoluminescence (PL) spectra of (a) pristine CsPbI_3_ PQDs without the peapod capping and (b) PbSO_4_-oleate-capped CsPbI_3_ PQDs. Photoluminescence quantum yields of the pristine CsPbI_3_ PQDs and the capped CsPbI_3_ PQDs are in (**B**). PbSO_4_-oleate capping reaction time was 60 min.

We first investigated the impact of PbSO_4_-oleate coverage on the fundamental photophysical and material properties of the CsPbX_3_ PQDs. Following the experimental procedure reported by Kamat et al. [18,19] we proceeded with the postsynthetic PbSO_4_-oleate coverage procedure following the synthesis of the CsPbBr_3_/CsPbI_3_ PQDs through a hot-injection process (see details in Materials and Methods). As shown in Figure 1 (for CsPbI_3_ PQDs) and Figure S1 (for CsPbBr_3_ PQDs), the PbSO_4_-oleate coverage did not affect the absorption bands of the CsPbBr_3_ and CsPbI_3_ PQDs. In addition, the positions of the emission peak maxima of the CsPbBr_3_ and CsPbI_3_ PQDs were unaffected by PbSO_4_-oleate capping. However, the PbSO_4_-oleate coverage led to an elongated absorption tail for both the CsPbBr_3_ and CsPbI_3_ PQDs. Notably, PbSO_4_-oleate formed a transparent layer with a peapod-like morphology on top of the PQDs; hence, the elongated absorption tails could be attributed to either (i) the scattering of the incident beam from the UV-Vis. absorption spectrometer or (ii) the generation of Urbach tails. Upon comparing the PLQYs of the PQDs with and without PbSO_4_-oleate capping, a decrease in the PLQYs (from 92.2% (with capping) to 58.7% (without capping) for CsPbI_3_ PQDs and from 45.6% (with capping) to 15.3% (without capping) for CsPbBr_3_ PQDs) was observed for all PQDs. This indicates that capping increases the number of defect sites in the PQDs. In addition, we observed that the capped PQD solution became blurry, in contrast to the solution of the PQDs without capping. This means that the capped PQDs scatter the incident light. Both scattering and the Urbach effect could affect the absorption characteristics even when the absorption band edges are identical. A decrease in PLQY after a transformation from initial ligand coverage to alternative ligands or surface moieties is commonly observed in the conventional ligand exchange process; this is owing to ligand cleavage on the surface of PQDs [22]. Note that there were no extra PL peaks except for the emission corresponding to the radiative recombination of the excited electrons from the CBM to the VBM. The symmetrical emission peaks indicate that there is no extra emission from any of the defect-mediated energy levels, which can be observed in conventional quantum dots [21]. In addition, as shown in Figure S2 (without baseline-corrected spectra owing to scattering), the effect of scattering increases the overall absorbance in all ranges beyond the band edge absorption. The baseline upshift reveals artificial beam scattering due to the capping layer. Furthermore, the Urbach energies corresponding to the elongated absorption tails could be obtained based on previous reports [14,23,24] and additional derivations (see Figure S3, Supplementary Note S1, and Table S1). In summary, both an increase in temperature and PbSO_4_-oleate coverage on the PQDs led to an increase in the Urbach energies. Considering that the ligands attached to the PQDs are in dynamic adsorption–desorption equilibria in the dispersed solution, increasing the temperature could facilitate both the adsorption and desorption of the ligands; therefore, we speculate that the overall surface defects on the PQDs increased. Therefore, the Urbach energies could be increased by increasing the solution temperature from 288 K to 318 K. Moreover, we inferred that the increase in the Urbach energies after the PbSO_4_-oleate coverage was due to ligand exchange from the OA/OAm to the PbSO_4_-oleate.

**Figure 2:**
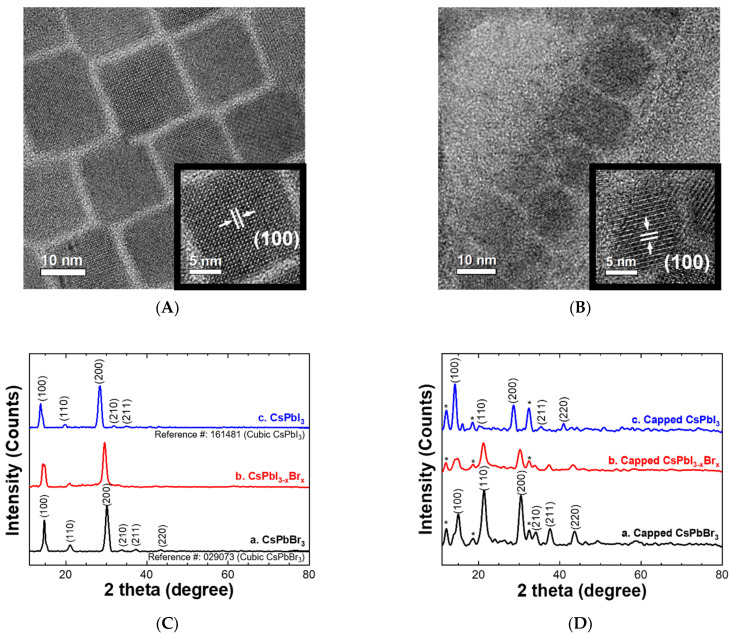
(**A**,**B**) TEM images of (**A**) pristine CsPbI_3_ PQDs without PbSO_4_-oleate coverage and (**B**) capped CsPbI_3_ PQDs with PbSO_4_-oleate coverage; coverage reaction time was 60 min (inset: enlarged CsPbI_3_ PQDs with (100) lattice fringe). (**C**,**D**) XRD patterns of CsPbX_3_ PQDs (**C**) without PbSO_4_-oleate coverage on PQDs and (**D**) with coverage.

Morphological TEM images of the capped and noncapped CsPbI_3_ PQDs are shown in Figure 2A,B, respectively. In the presence of PbSO_4_-oleate around the PQDs, the oleate carbon chain blurred the overall resolution of the PQDs because the carbon chain coverage induced carbon growth under e-beam exposure. Even though such blurred images could hinder the observation of the PQD edges, cubic-shaped CsPbI_3_ and the (100) plane (insets in Figure 2A,B) corresponding to cubic-crystallized (reference PDF number: 161481) CsPbI_3_ perovskites were identified from the HR-TEM images. Similar to observations made in previous studies [18,19] a peapod-like morphology could be observed after the capping process, confirming the successful PbSO_4_-oleate coverage of the PQDs. Furthermore, to investigate the effect of the crystalline structure of the PQDs on the PbSO_4_-oleate coverage, we obtained XRD patterns of the CsPbBr_3_/CsPbI_3_ PQDs with and without capping (see Figure 2C,D). In addition, by incorporating the two types of PQDs in the same solution and stirring under inert conditions until halide exchange occurred (overnight), we monitored the XRD patterns to observe the change in crystallinity. Notably, the XRD peaks corresponding to the cubic structure of the PQDs were observed in both cases, confirming that there was no dependence on the capping layer. Furthermore, the XRD patterns corresponding to PbSO_4_-oleate were observed in addition to the major XRD patterns. After the halide exchange, as shown in Figure S4, the formation of CsPbBr_x_I_3−x_ mixed halide PQDs occurred, and the corresponding patterns appeared between the peaks exhibited by the CsPbBr_3_/CsPbI_3_ PQDs. The variations in the XRD peak patterns for mixed halide perovskites with different halide compositions have been reported elsewhere [25,26]. Note that the peak position corresponding to the mixed halide perovskite was not centered between the peaks from the CsPbBr_3_/CsPbI_3_ PQDs because the concentrations of the CsPbBr_3_ and CsPbI_3_ PQDs were different (257 ± 15 nM and 428 ± 37 nM, respectively); further, the XRD peak movement was not proportional to the halide composition, in accordance with the empirical quadratic equation [27]. In addition, as shown in Figure S5, we elevated the temperature of the PQD solution; consequently, the peaks from the PQDs were maintained, with the appearance of additional peaks from PbSO_4_-oleate clusters. Furthermore, we were not able to notice distinct differences in the PQDs before and after the XRD measurements through direct visual observations; however, through TEM measurements, we noticed that long-term e-beam exposure damaged the PbSO_4_-oleate coverage as well as the PQDs. Thus, we concluded that X-ray/electron-beam irradiation could damage the PQDs, even though such damage could not be observed through human eyes. Note that details such as halide vacancies [28] and self-trapped holes [29] could not be determined using this XRD technique but could be tracked through spectroscopic tools. In short, we demonstrated that capping and solution temperature had no significant crystalline effect on the CsPbX_3_ PQDs.

### 3.2. Effect of PbSO_4_-Oleate Capping on Halide Exchange Kinetics between CsPbBr_3_ and CsPbI_3_ PQDs

**Figure 3:**
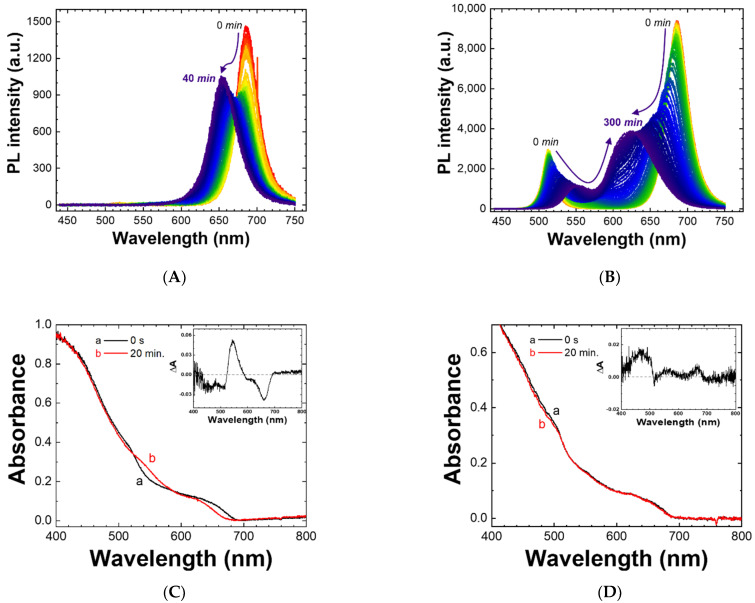
(**A**,**B**) In situ photoluminescence spectra of the halide exchange process among CsPbI_3_ and CsPbBr_3_ PQDs at 288 K (**A**) without PbSO_4_-oleate coverage on PQDs and (**B**) with PbSO_4_-oleate coverage on PQDs. (**C**,**D**) In situ absorption spectra for the halide exchange process among CsPbI_3_ and CsPbBr_3_ PQDs (**C**) without PbSO_4_-oleate coverage on PQDs and (**D**) with PbSO_4_-oleate coverage on PQDs. Initial absorbance (a, 0 s) and absorbance during halide exchange (b, 20 min) (inset: ∆A between a and b). Note that absorbance measurements were performed intermittently for shorter periods owing to the data collection and storage processing time required by the experimental setup.

We tracked the in situ emission and absorption changes during the halide exchange process among the CsPbBr_3_/CsPbI_3_ PQDs with and without PbSO_4_-oleate capping. Figure 3 shows the significantly delayed emission and absorption changes corresponding to the two capped PQDs compared to those of pristine PQDs (without PbSO_4_-oleate coverage). Thus far, we have elucidated that the PbSO_4_-oleate coverage did not have a considerable impact on the absorption/emission peak changes and structure of the PQDs. Nevertheless, as shown by the changes in the emission and absorption spectra in Figure 3 and the corresponding kinetics in Figure S6, notably retarded emission was observed in the case of the two capped PQDs mixed in the same solution. Note that the early kinetics could be influenced by ultrafast energy transfer from the CsPbBr_3_ PQDs to the CsPbI_3_ PQDs due to the ligands with long 18-carbon chains (OA, OAm) providing an appropriate interparticle distance for Föster resonance energy transfer (see Figure S7) [21,30,31]. The quenched emissions from the CsPbBr_3_ PQDs interfered with the tracking of peak intensity/position changes as the emissions became indiscernible from noise (see Figure S8). In addition, convection could occur in the solution during the hot-injection process for mixing the two PQDs using micropipettes. Thus, we observed the spectroscopic change kinetics after the mechanical convection ceased. Generally, the emission changes were more significant than the absorption changes. We propose that undesirable phenomena, such as scattering, reflection, and/or refraction, affected the measurements; therefore, the signal-to-noise ratio was higher for the absorption measurements than that for the emission measurements. We tracked the emission/absorption changes of the CsPbI_3_ PQDs. By considering first-order reaction kinetics based on the reports by Kamat et al. [18,19] we obtained similar results using both the emission and absorption changes, namely, a decrease in the halide exchange rate constants upon the application of the capping process. Particularly, as determined by emission and absorption measurements, the exchange rate constants decreased by 4.38% and 51.7%, respectively. Note that the emission changes shown in Figure 3A,B are more distinct than the absorption changes shown in Figure 3C,D with the insets. However, overall, the rate constants corresponded to similar orders; therefore, the overall kinetic traces and fitting results are considered to be reliable. Upon comparing the PQD core coverage for two different cases (OA/OAm vs. PbSO_4_-oleate), it is clear that the PbSO_4_-oleate coverage retarded the halide exchange process substantially. The halide exchange in the PQD-dispersed solution is mediated through the desorption/adsorption of ligands upon exposure of the PQD surface; thus, we propose that PbSO_4_-oleate coverage hinders this desorption/adsorption, thereby deteriorating the rate of the adsorption/desorption process. We also attempted second- or zeroth-order reaction kinetics fitting, but considering that (i) the activation energy for iodide, a major element entailed in halide migration, is lower than that for bromide [10,13] and that (ii) I-Pb binding constants to lead complexation is about one-seventh times lower than that of the Br-Pb binding constants [26,32], we followed first-order fitting for this halide exchange kinetics.

**Figure 4:**
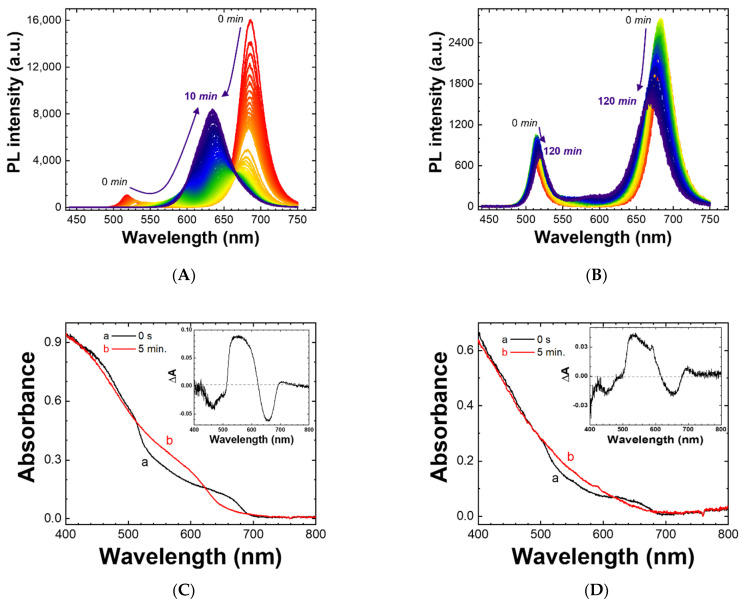
(**A**,**B**) In situ photoluminescence spectra of the halide exchange process among CsPbI_3_ and CsPbBr_3_ PQDs at 318 K (**A**) without PbSO_4_-oleate coverage on PQDs and (**B**) with PbSO_4_-oleate coverage on PQDs. (**C**,**D**) In situ absorption spectra for the halide exchange process among CsPbI_3_ and CsPbBr_3_ PQDs (**C**) without PbSO_4_-oleate coverage on PQDs and (**D**) with PbSO_4_-oleate coverage on PQDs. Initial absorbance (a, 0 s) and absorbance during halide exchange (b, 5 min.) (inset: ∆A between a and b). Note that absorbance measurements were performed intermittently for shorter periods owing to the data collection and storage processing time required by the lab-built IPCE-CCD setup.

To study the effect of temperature on the halide exchange kinetics and to obtain the activation energies with variations in the PQD solution temperatures, the halide exchange processes were monitored through two in situ emission/absorption spectroscopies. Representative emission/absorption spectral changes are presented in Figure 4, and their kinetics are shown in Figure S9. In addition, we investigated the overall kinetic changes by varying the capping reaction times (5 min, 30 min, and 60 min) to confirm the effect of PbSO_4_-oleate coverage on the halide exchange kinetics. The detailed rate constants are presented in Table S2. In short, an increase in temperature led to an increase in the halide exchange reaction kinetics, but interestingly, the rate constants increased more drastically in the case of the capped PQDs. Based on both spectroscopic measurements, the rate constant increment for capped PQDs was approximately 2.7 times higher than that for the PQDs without capping. The rate constant enhancements at higher temperatures and their comparisons are elucidated as follows.

(2) $$\left(\mathrm{Rate}\;\mathrm{constant}\;\mathrm{enhancements}\right)=\left(\;\frac{k\;for\;capped\;PQDs\;at\;318\;K}{k\;for\;capped\;PQD\;at\;288\;K}/\frac{k\;for\;pristine\;PQDs\;at\;318\;K}{k\;for\;pristine\;PQDs\;at\;288\;K}\right)$$

The drastic increments in the rate constants ($\frac{∆k_{318K}}{∆k_{288K}}$) are reflected by increasing slopes given by the Arrhenius equation ($\frac{∆lnk}{∆T^{-1}}$); therefore, it was expected that equation (2) would yield a higher *E_a_* for capped PQDs than that for the pristine PQDs. Furthermore, a distinct ∆A was observed in the absorption difference spectra after the halide exchange between the two PQDs at 318 K (two insets in Figure 4C,D), in contrast to the ∆A at 288 K (see insets in Figure 3C,D). The distinct increase in temperature facilitated the halide exchange process, as expected based on the Arrhenius equation. We speculate that the dynamical desorption/adsorption of OA/OAm or PbSO_4_-oleate coverage could be facilitated by increasing the temperature of the PQD-dispersed solution. The excitation spectra of the CsPbBr_3_/CsPbI_3_ PQDs with respect to (i) PbSO_4_-oleate coverage, (ii) temperature, and (iii) the halide exchange process are shown in Figure S10. Notably, distinct emissions around the band edge could be observed for all (CsPbBr_3_/CsPbBr_x_I_3-x_/CsPbI_3_) PQDs without PbSO_4_-oleate coverage at lower temperatures. By contrast, less distinct emissive features around the band edge were observed for all (CsPbBr_3_/CsPbBr_x_I_3-x_/CsPbI_3_) capped PQDs. We attribute this to a decrease in the PLQY after the capping process and to higher Urbach energies obtained with an increase in temperature, as shown in Figure 1 and Figures S1–S3, and Table S1.

**Figure 5:**
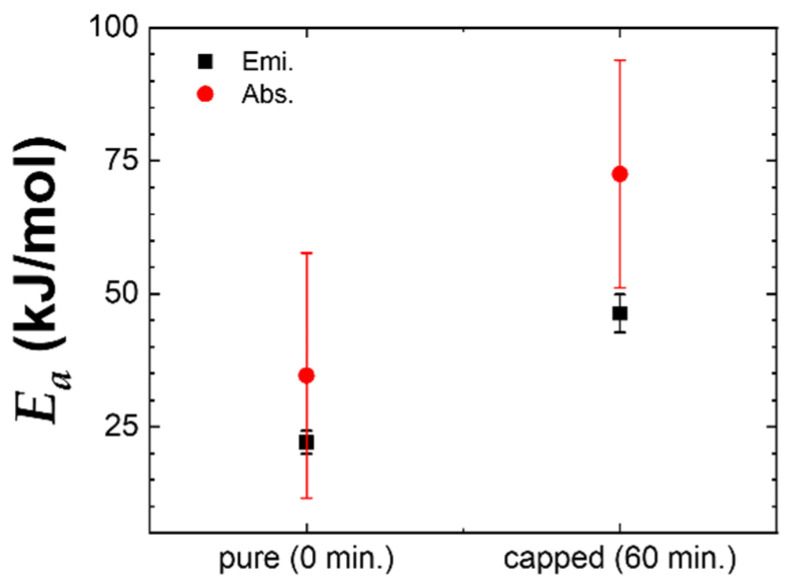
Activation energies obtained through the Arrhenius equation with linear fitting (*lnk* vs. *1/T*). Two different spectroscopies (emission and absorption) were performed to obtain *E_a_*. The *E_a_* values are presented with standard deviations for the pristine and capped QDs.

**Scheme 1:**
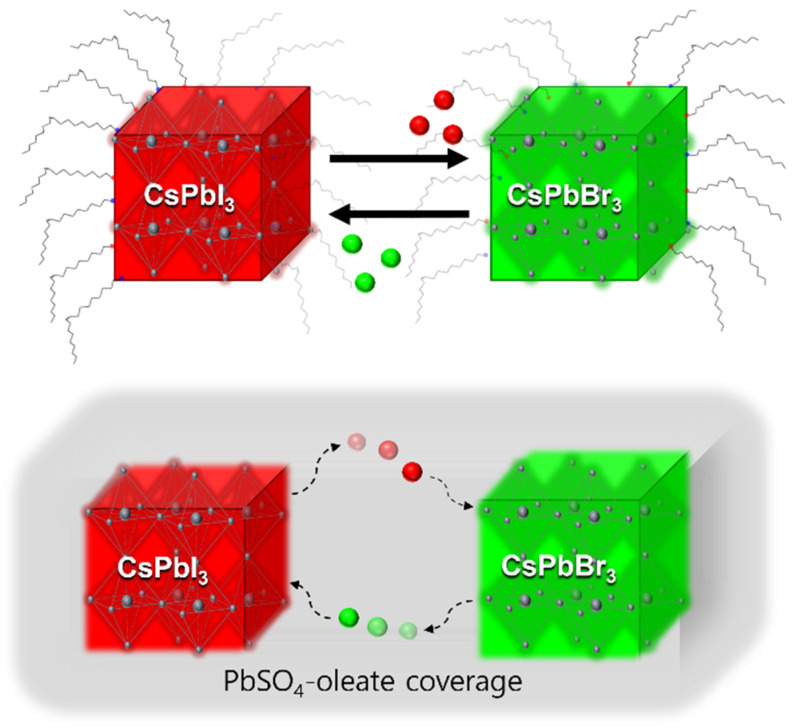
Schematics of (**top**) halide exchange among pristine CsPbI_3_ and CsPbBr_3_ PQDs and (**bottom**) PbSO_4_-oleate covered CsPbI_3_ and CsPbBr_3_ PQDs.

We plotted *lnk* vs. *1/T* to obtain *E_a_* using the Arrhenius equation. In Figure S11, the detailed plots are presented. Note that we repeated the absorption measurements to obtain *E_a_* as the signal-to-noise ratio of the absorption measurements was lower than that of the emission measurements (see details in Table S2). Figure 5 shows the changes in *E_a_* for the halide exchange process among the CsPbBr_3_/CsPbI_3_ PQDs depending on PbSO_4_-oleate capping. As expected from the emission/absorption spectra in Figure 3 and Figure 4 and the significantly controlled halide exchange process for the PbSO_4_-oleate-capped PQDs [18,19], capping increased the activation energies for the halide exchange among the CsPbBr_3_/CsPbI_3_ PQDs. The overall values are presented in Table 1, with the Arrhenius plots shown in Figures S11 and S12. In the case of the pristine PQDs, the *E_a_* values obtained through emission measurements and absorption measurements were 22.07 ± 2.38 kJ/mol (0.23 ± 0.02 eV) and 34.62 ± 23.08 kJ/mol (0.36 ± 0.24 eV), respectively. The obtained values are similar to previously reported values [33] (44 kJ/mol or 0.46 eV). Note that the A-site cation exchange process (e.g., FA^+^ and Cs^+^) has a higher *E_a_* value [34] (63 kJ/mol or 0.65 eV) than that of the halide exchange process. The values correspond, within the standard deviations; moreover, they are similar to the halide migration activation energies reported for perovskites in the literature [10,13]. By contrast, after the postsynthetic PbSO_4_-oleate capping process, *E_a_* increased by 2.10 times (through emission measurements) or 2.56 times (through absorption measurements). The values obtained through the emission and absorption measurements and the corresponding increasing trends were similar. Furthermore, by controlling the PbSO_4_-oleate capping reaction time, we obtained activation energies for the halide exchange process beyond the capping layer. The enhanced *E_a_* demonstrated that PbSO_4_-oleate coverage can significantly retard halide exchange among the CsPbBr_3_/CsPbI_3_ PQDs. In addition, based on the peapod-like morphology shown in Figure 2B and the kinetics shown in Figure 3 and Figure 4, we concluded that PbSO_4_-oleate coverage retarded the desorption/adsorption of the conventional ligands, thereby requiring more energy to induce the halide exchange process. Based on these results, the overall halide exchange processes with and without PbSO_4_-oleate coverage are schematically illustrated in Scheme 1. The coverage could interfere with the halide exchange process owing to the higher *E_a_* and increased halide migration pathways.

## 4. Conclusions

In conclusion, we demonstrated the role of the PbSO_4_-oleate capping of CsPbI_3_/CsPbBr_3_ PQDs in suppressing halide exchange. Postsynthetic PbSO_4_-oleate capping could introduce defective sites in the PQDs, along with an absorption tail, and lower the PLQY; however, the absorption band edge, emission peak position corresponding to the band gap, and structural properties of the PQDs were maintained. After the PbSO_4_-oleate capping, the halide exchange kinetics were retarded; furthermore, the temperature dependence of the halide exchange kinetics enabled us to obtain various activation energies for the process based on in situ UV-Vis. absorption and PL spectroscopy. The observed increase in activation energies with increased PbSO_4_-oleate coverage on the PQDs demonstrated that the halide exchange between the CsPbI_3_/CsPbBr_3_ PQDs is suppressed. These results can be attributed to the maintenance of the original photophysical properties of the PQDs in the mixed state. This study can contribute to the development of alternative structural designs for white LEDs, photovoltaic cells, and photocatalysts.

## Figures and Tables

**Table 1:** Activation energies obtained through in situ emission/absorption spectroscopies depending on the postsynthetic PbSO_4_-oleate capping process.

*E_a_* from Different Spectroscopies (kJ/mol or eV)	Noncapped	5 min	30 min	60 min
Emission	22.07 ± 2.38 kJ/mol (0.23 ± 0.02 eV)	54.53 ± 18.94 (0.57 ± 0.05 eV)	55.30 ± 4.41 (0.57 ± 0.05 eV)	46.31 ± 3.57 (0.48 ± 0.04 eV)
Absorbance	34.62 ± 23.08 (0.36 ± 0.24 eV)	-	-	88.70 ± 20.09 (0.92 ± 0.21 eV)

## Data Availability

Data are contained within the article.

## Supplementary Materials

The following are available online at https://www.mdpi.com/article/10.3390/nano11102515/s1, Supplementary Note S1: Calculation of the Urbach energies; Figure S1: Normalized UV-Vis. absorption spectra and PL spectra of CsPbBr3; Figure S2: Normalized UV-Vis. absorption spectra of CsPbI3 (without baseline correction) and CsPbBr3 PQDs; Figure S3: Optical absorption spectra of the CsPbX3 PQDs; Figure S4: Enlarged XRD patterns of the CsPbBr3, CsPbI3, and CsPbI3-xBrx PQDs; Figure S5: XRD patterns of various PQDs at different temperatures; Figure S6: Kinetic fitting results of halide exchange processes; Figure S7: In situ photoluminescence spectra of CsPbBr3 (~12 s); Figure S8: In situ photoluminescence spectra of CsPbBr3 PQDs, enlarged spectra for the emission from the CsPbBr3 PQDs; Figure S9: Kinetic fitting results of halide exchange processes with varying temperatures; Figure S10: Excitation spectra of the CsPbX3 PQDs under divalent conditions; Figure S11: Arrhenius plots of ln kmix versus 1/T (PbSO4-oleate coverage 0min and 60min); Figure S12: Arrhenius plots of ln kmix versus 1/T (PbSO4-oleate coverage 5min and 30min); Table S1: Obtained Urbach energies; Table S2: Halide exchange reaction rate constants according to temperature and PbSO_4_-oleate reaction times.
