# Supplementary material for: Effect of PbSO4-Oleate Coverage on Cesium Lead Halide Perovskite Quantum Dots to Control Halide Exchange Kinetics
Source: Nanomaterials (Basel). 2021 Sep 27;11(10):2515. doi: 10.3390/nano11102515 (PMC8538890; doi:10.3390/nano11102515)
Supplement: Supplementary file 1 [file nanomaterials-11-02515-s001.zip › nanomaterials-1382342-supplementary.pdf]

Supplementary Material

Effect of $PbSO_4$-Oleate Coverage on Cesium Lead Halide Perovskite Quantum Dots to Control Halide Exchange Kinetics

Yeonsu Woo, Seeun Park and Seog Joon Yoon *

Department of Chemistry, College of Natural Science, Yeungnam University, 280 Daehak-Ro, Gyeongsan-si, Gyeongsangbuk-do, Korea; dndustn19@naver.com (Y.W.); qqkrtp1220@naver.com (S.P.)
* Correspondence: yoon@yu.ac.kr

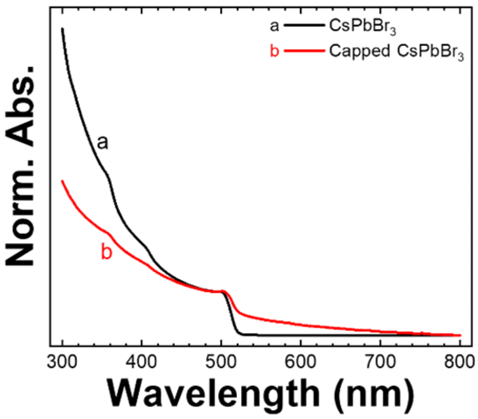

(**A**)

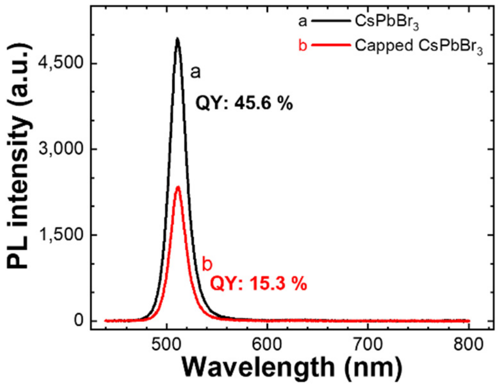

(**B**)

Figure S1. (A) Normalized UV-Vis. absorption spectra (Normalized absorbance at band edge) of (a) pristine $CsPbBr_3$ PQDs without the peapod capping and (b) $PbSO_4$-oleate capped-$CsPbBr_3$ PQDs. **(B)** Photoluminescence (PL) spectra of (a) pristine $CsPbBr_3$ PQDs without the peapod capping and (b) $PbSO_4$-oleate capped-$CsPbBr_3$ PQDs. Photoluminescence quantum yields of the pristine $CsPbBr_3$ PQDs and the capped $CsPbBr_3$ PQDs were in **(B)**. $PbSO_4$-oleate capping reaction time was 60 min.

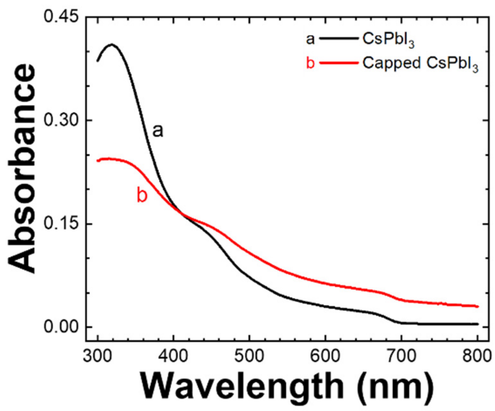

(**A**)

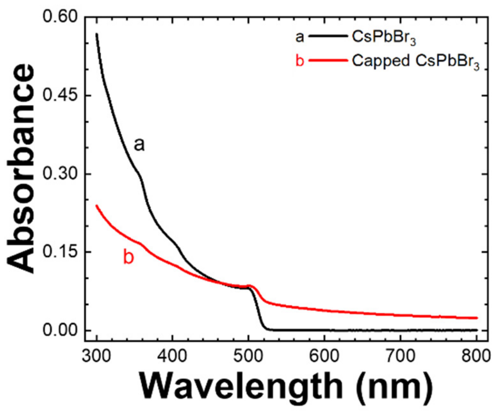

(**B**)

Figure S2. UV-Vis. absorption spectra of PQDs with and without $PbSO_4$-oleate coverage, presenting with baseline up-shift due to light scattering from the $PbSO_4$-oleate capping layer. **(A)** UV-Vis. absorption spectra of (a) pristine $CsPbI_3$ PQDs without the peapod capping and (b) $PbSO_4$-oleate capped-$CsPbBr_3$ PQDs. **(B)** UV-Vis. absorption spectra of (a) pristine $CsPbBr_3$ PQDs without the peapod capping and (b) $PbSO_4$-oleate capped-$CsPbBr_3$ PQDs. $PbSO_4$-oleate capping reaction time was 60 min.

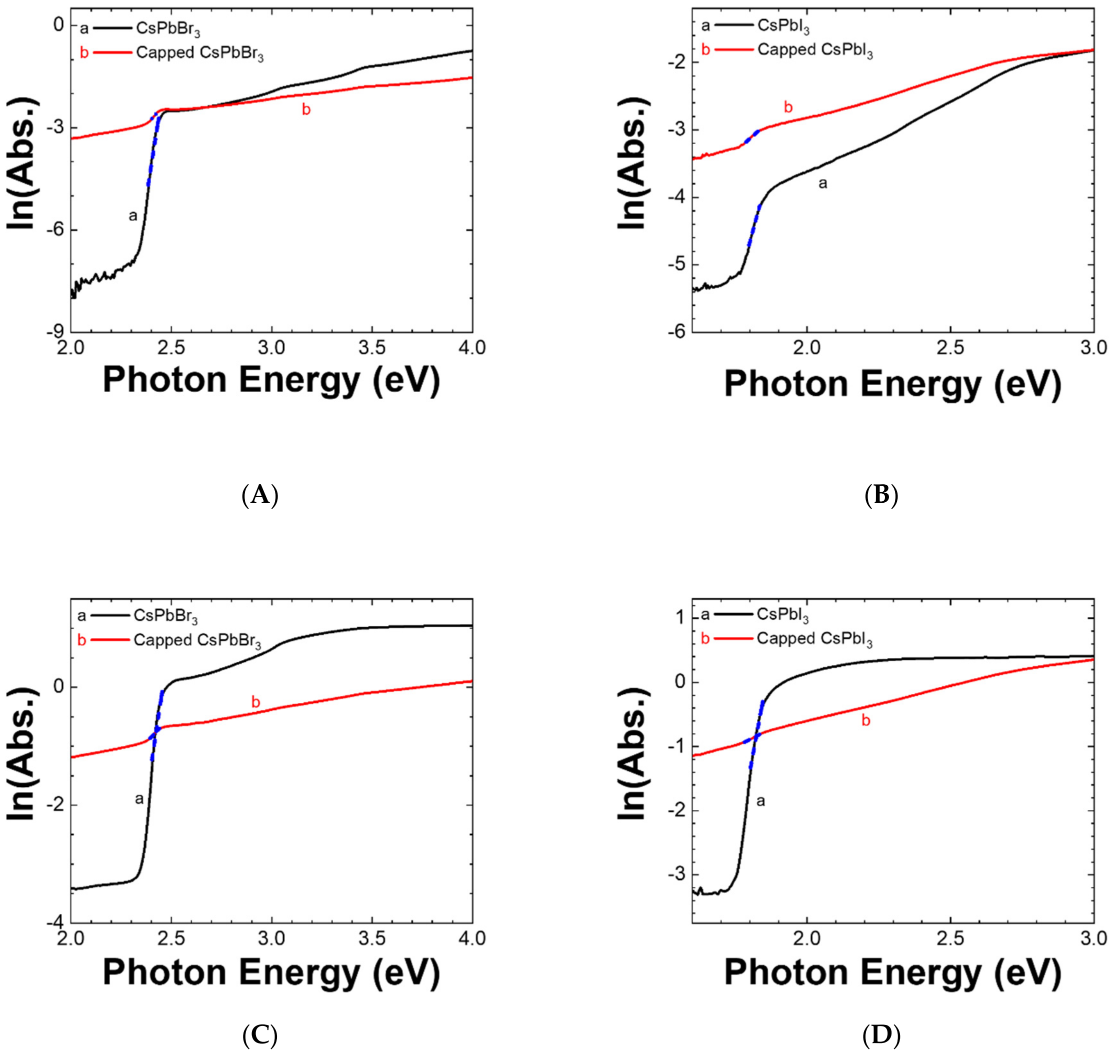

Figure S3. Optical absorption spectra of the $CsPbX_3$ (**A,C**: $CsPbBr_3$, **B,D**: $CsPbI_3$) PQDs with and without the $PbSO_4$-oleate capping under different temperatures (**A,B**: 288K, **C,D**: 318K). Urbach energy was calculated through linear fitting of the Urbach tail (blue dashed line in the spectra). The spectra were represented through UV-Vis. absorption spectra with converting wavelength to photon energy in x-axis and natural log of the absorption in y-axis.

Calculation of Urbach energy (E_U) from absorption spectrum of the PQDs

Assuming that the absorption coefficient(α) is proportional to absorbance (A)/film thickness(d):

$$\alpha = \ln 10 \times \frac{A}{d} \tag{S1}$$

The Urbach energy (E_U) can be obtained the absorption tail with the form as described earlier,[1-3] as follows:

$$\alpha = \alpha_0 \exp(\frac{E - E_0}{E_U}) \tag{S2}$$

E represents photon energy. Applying Equation (S1) to Equation (S2), the Equation (S2) can be described as follows:

$$\ln 10 \times \frac{A}{d} = \ln 10 \times \frac{A_0}{d} \times \exp(\frac{E - E_0}{E_U}) \tag{S3}$$

The Equation (S3) can be simplified. The Equation (S3) can be rearranged, as follows:

$$\ln A = \ln A_0 + \frac{1}{E_U}(E - E_0) \tag{S4}$$

$$\ln A = \frac{1}{E_U}E - \frac{1}{E_U}E_0 + \ln A_0 \tag{S5}$$

Through linear fitting at the absorption tail (*lnA vs. E*), the *Eu* were determined through 1/(slope). The Urbach energies are summarized in Table S1.

Table S1. Summarized Urbach energies obtained through optical absorption spectra shown in Figure S3.

E_u from different PQDs in various temperatures (K)	Non-capped $CsPbBr_3$ (meV)	Capped $CsPbBr_3$ (meV)	Non-capped $CsPbI_3$ (meV)	Capped $CsPbI_3$ (meV)
288 K	26.7 ± 2.3	148.8 ± 6.4	63.8 ± 2.0	267.7 ± 7.7
318 K	44.2 ± 5.0	286.4 ± 11.5	41.3 ± 2.1	437.8 ± 4.6

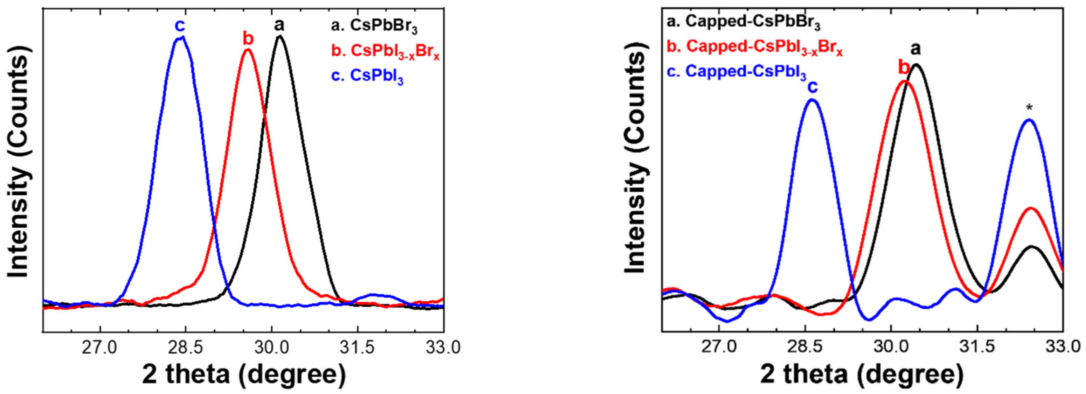

(**A**) (**B**)

Figure S4. (A) Enlarged XRD patterns in between 26° ~ 33° for (200) peaks for the **(a)** pristine $CsPbBr_3$ **(b)** $CsPbI_{3-x}Br_x$ **(c)** $CsPbI_3$ PQDs. **(B)** The enlarged XRD patterns for (200) peaks for the $PbSO_4$-oleate capped PQDs: **(a)** $CsPbBr_3$ **(b)** $CsPbI_{3-x}Br_x$ **(c)** $CsPbI_3$ (" * " sign indicates peak from the $PbSO_4$-oleate capping layer). $PbSO_4$-oleate capping reaction time was 60 min.

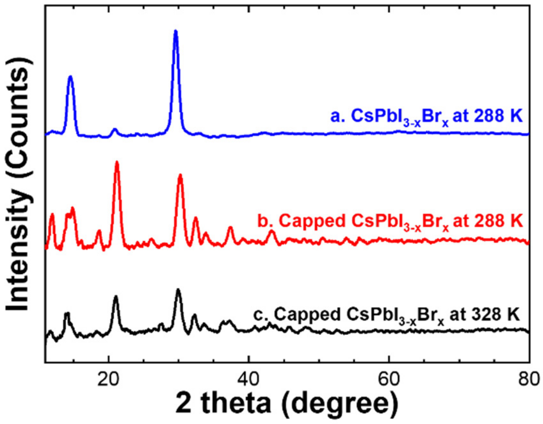

Figure S5. XRD patterns of two $CsPbI_3$ and $CsPbBr_3$ PQDs mixture after completion of halide exchange process with and without $PbSO_4$-oleate coverage, and under divalent reaction temperatures: **(a)** mixing both pristine $CsPbI_3$, and pristine $CsPbBr_3$ PQDs, **(b,c)** $PbSO_4$-oleate covered $CsPbI_{3-x}Br_x$ **(b)** at 288 K and **(c)** 328 K. $PbSO_4$-oleate capping reaction time was 60 min.

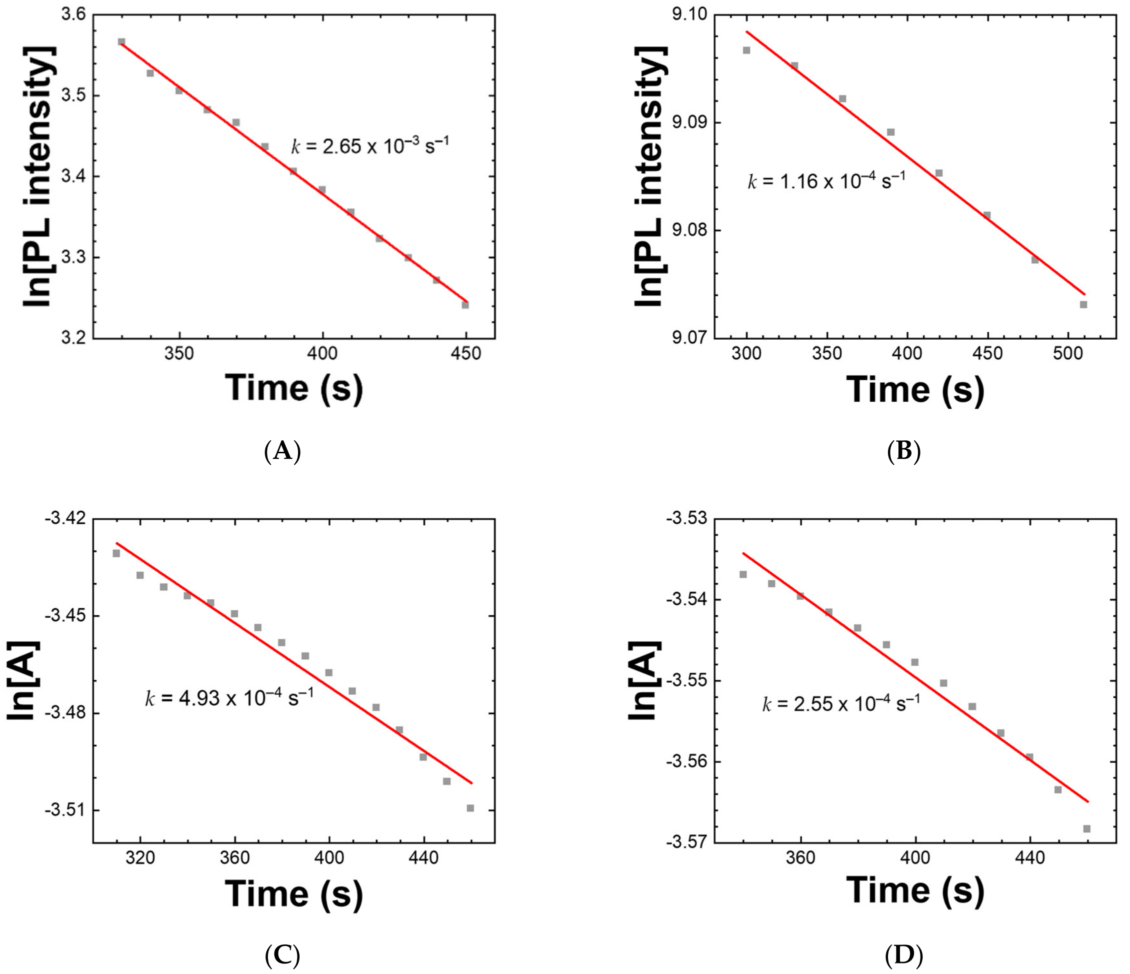

Figure S6. (A,B) At 288 K, with linear fitting, plots of ln(emission intensity) vs. time from emission at initial peak maximum while halide exchange processes. Kinetics while the process among **(A)** pristine $CsPbBr_3/CsPbI_3$ PQDs and **(B)** capped $CsPbBr_3/CsPbI_3$ PQDs. **(C,D)** With linear fitting, plots of ln(concentration of $CsPbI_3$ PQDs) vs. time from absorption band edge of the $CsPbI_3$ PQDs while halide exchange processes. Kinetics while the process among **(C)** pristine $CsPbBr_3/CsPbI_3$ PQDs and **(D)** capped $CsPbBr_3/CsPbI_3$ PQDs.

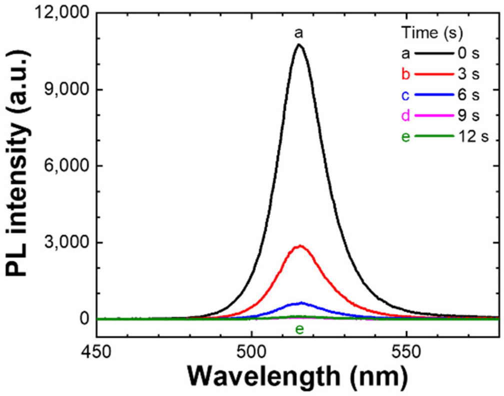

(a)

Figure S7. In situ photoluminescence spectra of $CsPbBr_3$ PQDs while the halide exchange process with $CsPbI_3$ PQDs at 288 K (Enlarged region for emission from $CsPbBr_3$ PQDs).

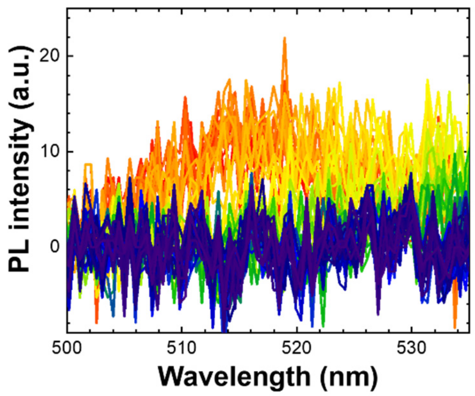

Figure S8. *in-situ* photoluminescence spectra of $CsPbBr_3$ PQDs while the halide exchange process with $CsPbI_3$ PQDs at 288 K (Enlarged region in between 500 to 535 nm from Figure 3A). Following red (initial spectrum) to blue (last spectrum for this set of time-based emission spectra) gradient color change (red to purple) present time-dependent emission change during entire measurement time.

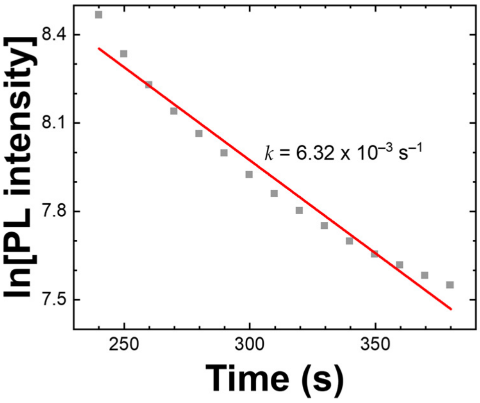

(**A**)

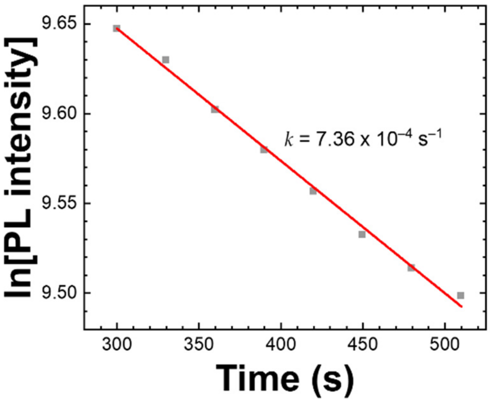

(**B**)

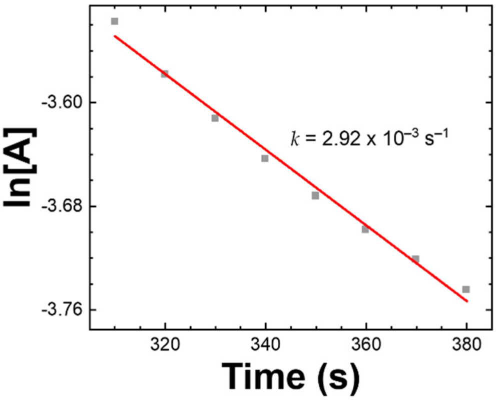

(**C**)

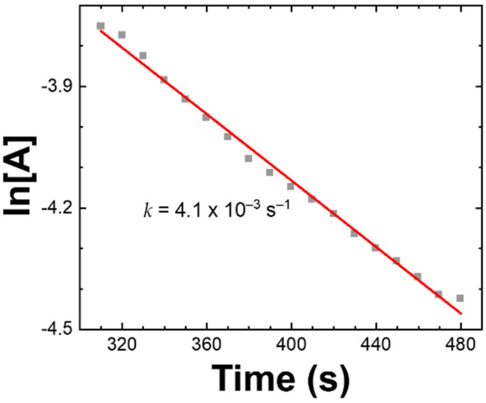

(**D**)

Figure S9. (A,B) At 318 K, with linear fitting, plots of ln(emission intensity) vs. time from emission at initial peak maximum while halide exchange processes. Kinetics while the process among **(A)** pristine $CsPbBr_3/CsPbI_3$ PQDs and **(B)** capped $CsPbBr_3/CsPbI_3$ PQDs. **(C,D)** With linear fitting, plots of ln(concentration of $CsPbI_3$ PQDs) vs. time from absorption band edge of the $CsPbI_3$ PQDs while halide exchange processes. Kinetics while the process among **(C)** pristine $CsPbBr_3/CsPbI_3$ PQDs and **(D)** capped $CsPbBr_3/CsPbI_3$ PQDs.

Table S2. Halide exchange reaction rate constants (k_{at}) with dependent on two factors: temperatures (288, 298, 208, and 318 K) and $PbSO_4$-oleate coverage reaction times (5 min. and 30 min.) on $CsPbX_3$ PQDs.

k_{at} at various temp. (K)		288	298	308	318
5 min. capping (s^{-1})	1st	$1.66 \times 10^{-4} \pm 1.18 \times 10^{-5}$	$1.01 \times 10^{-3} \pm 2.99 \times 10^{-5}$	$9.99 \times 10^{-4} \pm 2.69 \times 10^{-5}$	$1.76 \times 10^{-3} \pm 5.52 \times 10^{-5}$
	2nd	$3.07 \times 10^{-4} \pm 1.18 \times 10^{-5}$	$1.03 \times 10^{-3} \pm 4.85 \times 10^{-5}$	$6.73 \times 10^{-4} \pm 1.23 \times 10^{-5}$	$1.12 \times 10^{-3} \pm 3.24 \times 10^{-5}$
30 min. capping (s^{-1})	1st	$3.45 \times 10^{-4} \pm 6.62 \times 10^{-5}$	$8.98 \times 10^{-4} \pm 5.04 \times 10^{-5}$	$1.55 \times 10^{-3} \pm 4.64 \times 10^{-5}$	$2.92 \times 10^{-3} \pm 7.60 \times 10^{-5}$
	2nd	$2.18 \times 10^{-4} \pm 7.13 \times 10^{-6}$	$3.94 \times 10^{-4} \pm 2.71 \times 10^{-5}$	$9.28 \times 10^{-4} \pm 3.96 \times 10^{-5}$	$1.66 \times 10^{-3} \pm 1.12 \times 10^{-4}$
	3rd	$2.00 \times 10^{-4} \pm 9.37 \times 10^{-6}$	$4.05 \times 10^{-4} \pm 1.36 \times 10^{-5}$	$7.66 \times 10^{-4} \pm 3.89 \times 10^{-5}$	$2.20 \times 10^{-3} \pm 8.83 \times 10^{-5}$

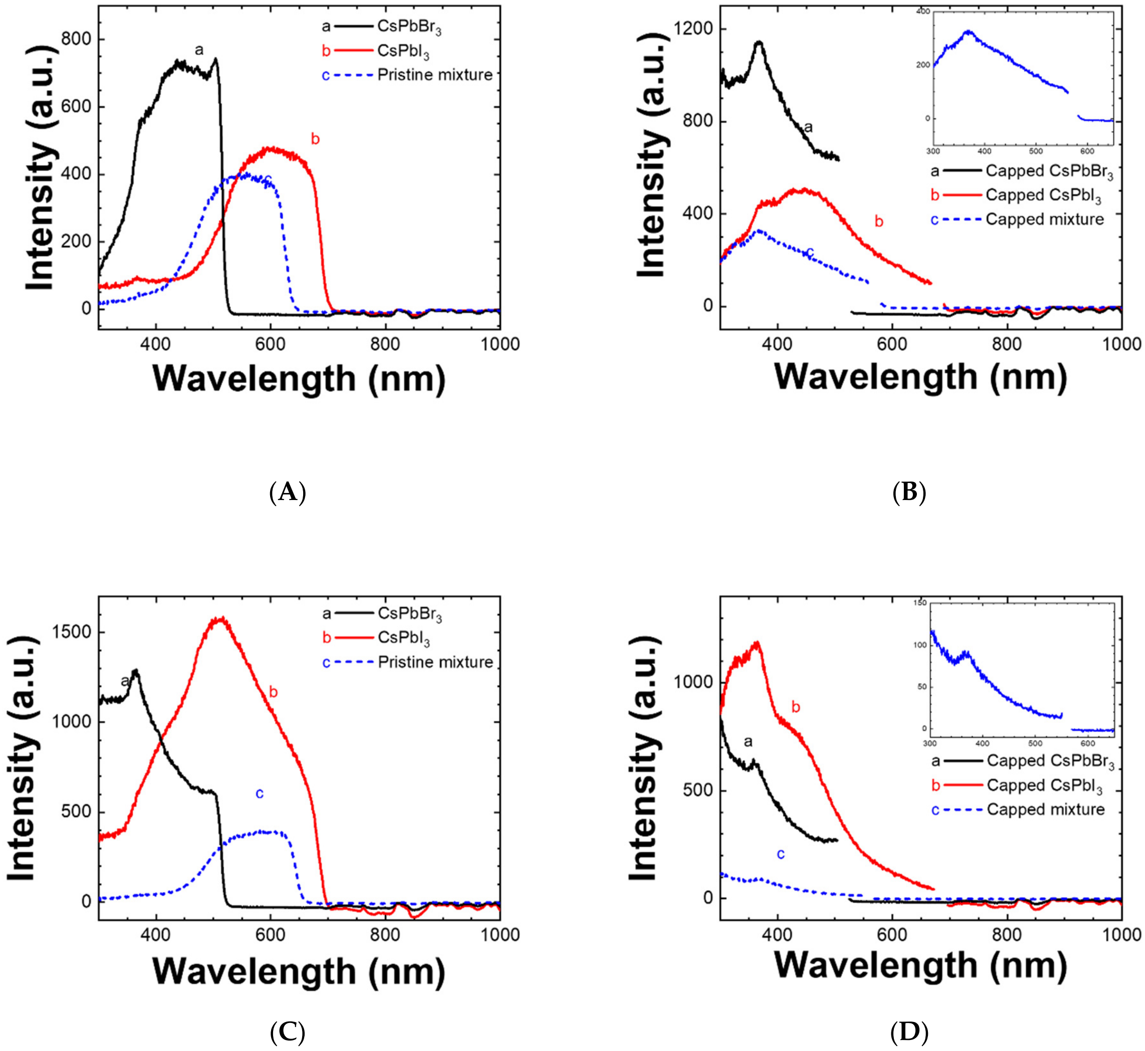

Figure S10. Excitation spectra of **(A)** pristine PQDs at 288 K, **(B)** capped PQDs at 288 K, **(C)** pristine PQDs at 318 K, **(D)** capped PQDs at 318 K. Note that dashed spectra were obtained after halide exchange between the $CsPbBr_3$/$CsPbI_3$ PQDs with insets to observe clearly. Artificial interferences from band edge emission from PQDs were disregarded in the spectra in **(B)** and **(D)**.

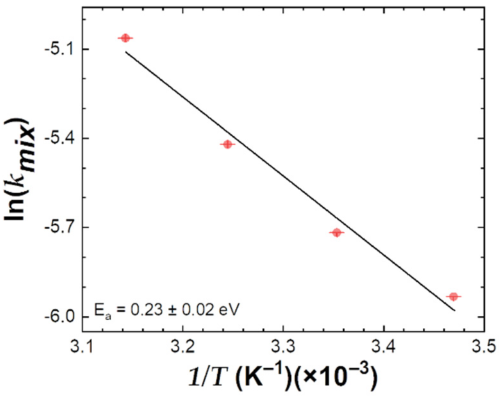

(A)

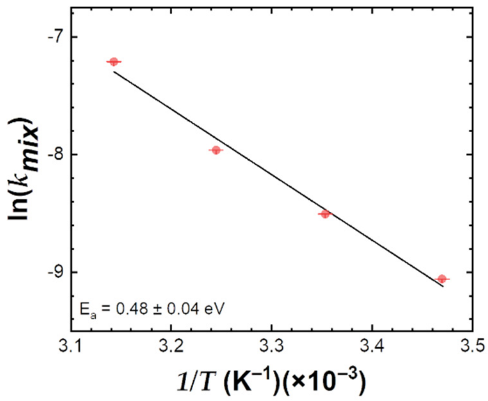

(B)

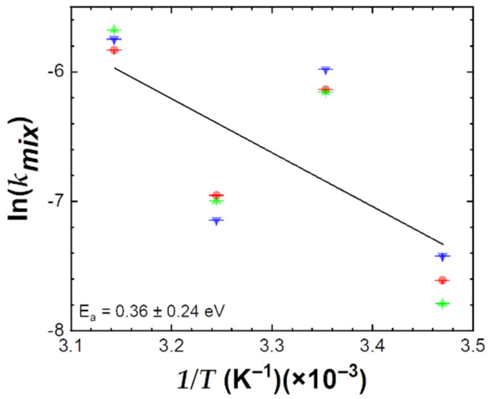

(C)

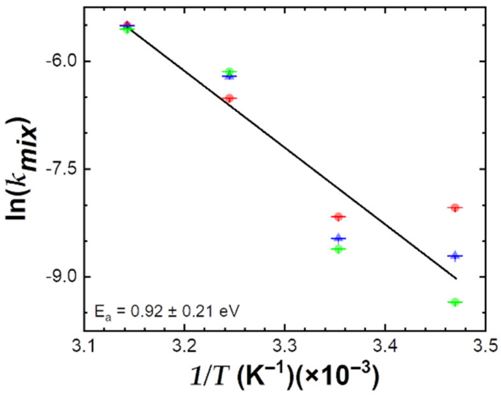

(D)

Figure S11. Arrhenius plots of ln k_{mix} versus 1/T for halide exchange process between $PbSO_4$-oleate covered $CsPbI_3$ and covered $CsPbBr_3$ PQDs obtained **(A,B)** emission spectroscopy and **(C,D)** absorbance spectroscopy with two different $PbSO_4$-oleate coverage reaction times: **(A,C)** 0 min (as pristine), **(B,D)** 60 min. The line is a linear fit to obtain the activation energies for the halide exchange reaction.

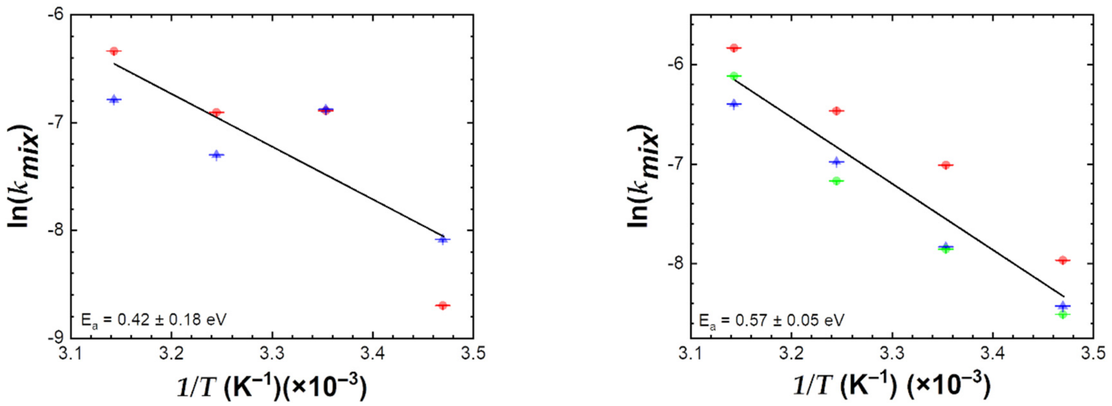

(**A**) (**B**)

Figure S12. (A,B) Arrhenius plots of ln k_{mix} versus 1/T for halide exchange process between $PbSO_4$-oleate covered $CsPbI_3$ and $CsPbBr_3$ PQDs with two different $PbSO_4$-oleate coverage reaction times: **(A)** 5 min, **(B)** 30 min. The line is a linear fit to obtain the activation energies for the halide exchange reaction.

References

1. Shen, Q.; Ogomi, Y.; Chang, J.; Toyoda, T.; Fujiwara, K.; Yoshino, K.; Sato, K.; Yamazaki, K.; Akimoto, M.; Kuga, Y.; et al. Optical Absorption, Charge Separation and Recombination Dynamics in Sn/Pb Cocktail Perovskite Solar Cells and their Relationships to Photovoltaic Performances. *J. Mater. Chem. A* **2015**, *3*, 9308–9316.
2. Jean, J.; Mahony, T.S.; Bozyigit, D.; Sponseller, M.; Holovský, J.; Bawendi, M.G.; Bulović, V. Radiative Efficiency Limit with Band Tailing Exceeds 30% for Quantum Dot Solar Cells. *ACS Energy Lett.* **2017**, *2*, 2616–2624.
3. Caselli, V.M.; Wei, Z.; Ackermans, M.M.; Hutter, E.M.; Ehrler, B.; Savenije, T.J. Charge Carrier Dynamics upon Sub-bandgap Excitation in Methylammonium Lead Iodide Thin Films: Effects of Urbach Tail, Deep Defects, and Two-Photon Absorption. *ACS Energy Lett.* **2020**, *5*, 3821–3827.
